# Refactoring the pikromycin synthase for the modular biosynthesis of macrolide antibiotics in E. coli

**DOI:** 10.21203/rs.3.rs-5640596/v1

**Published:** 2025-01-08

**Authors:** Adrian Keatinge-Clay, Takeshi Miyazawa

**Affiliations:** UT Austin

## Abstract

While engineering modular polyketide synthases (PKSs) using the recently updated module boundary has yielded libraries of triketide-pentaketides, this strategy has not yet been applied to the combinatorial biosynthesis of macrolactones or macrolide antibiotics. We developed a 2-plasmid system for the construction and expression of PKSs and employed it to obtain a refactored pikromycin synthase in *E. coli* that produces 85 mg of narbonolide per liter of culture. The replacement, insertion, deletion, and mutagenesis of modules enabled access to hexaketide, heptaketide, and octaketide derivatives. Supplying enzymes for desosamine biosynthesis and transfer enabled production of narbomycin, pikromycin, YC-17, methymycin, and 6 derivatives thereof. Knocking out pathways competing with desosamine biosynthesis and supplying the editing thioesterase PikAV boosted the titer of narbomycin 55-fold to 37 mgL^−1^. The replacement of the 3rd pikromycin module with its 5th yielded a new macrolide antibiotic and demonstrates how libraries of macrolide antibiotics can be readily accessed.

## INTRODUCTION

Antimicrobial resistance is an ever-increasing threat to human health. Consequently, there is a growing need to discover and develop new antibiotics^1^. Unfortunately, the pace of both discovering new antibiotics and developing antibiotics through medicinal chemistry has slowed^2^. Modular synthesis, through which diversity is created from relatively simple building blocks, can accelerate these processes^3^. A modular chemical synthesis recently yielded a library of > 300 macrolide antibiotic derivatives, 2 of which displayed superior potencies compared to those in clinical use^4^.

The modular syntheses of macrolide antibiotics, such as erythromycin, pikromycin, and tylosin, were first conducted by polyketide synthase (PKS) assembly lines within actinomyces bacteria^5–8^. These assembly lines are themselves modular, where each module (updated definition used^9,10^) adds one ketide monomer to a growing polyketide chain. They minimally contain 3 domains - an acyltransferase (AT), an acyl carrier protein (ACP), and a ketosynthase (KS). During the catalytic cycle of a module, ACP acquires the polyketide chain from the KS of the upstream module through an a-carboxyacyl extender unit obtained from AT, shuttles the extended chain to optional processing enzymes, such as ketoreductase (KR), dehydratase (DH), and enoylreductase (ER) domains, and to KS, which acquires properly processed chains through transacylation. The most downstream domain of the assembly line is most commonly a thioesterase (TE) that releases the polyketide chain through cyclization or hydrolysis.

Most efforts to engineer PKSs have been based on the traditional module boundary and have not been successful^10^. This boundary was defined immediately upstream of KS when the genes encoding the erythromycin PKS were sequenced in 1990, in analogy with the mammalian fatty acid synthase (FAS), an iterative PKS in which KS is the most upstream domain^11,12^. In 2017, genome-mining of modular PKSs that produce related aminopolyols helped reveal that the KSs of modular PKSs evolutionarily co-migrate with processing domains upstream of them and that the module boundary is actually immediately downstream of KS^9,10^. Indeed, synthases engineered with the updated module boundary consistently outperform synthases engineered with the traditional module boundary^13–15^. Our lab recently developed a BioBrick-like platform to rapidly assemble synthases on a single expression plasmid through the ligation of DNA fragments encoding updated modules. We employed it to combinatorially assemble the modules of the pikromycin synthase (*Streptomyces venezuelae* ATCC 15439) into 5 triketide, 25 tetraketide, and 125 pentaketide synthases and assess their function^16^.

**Figure 1:**
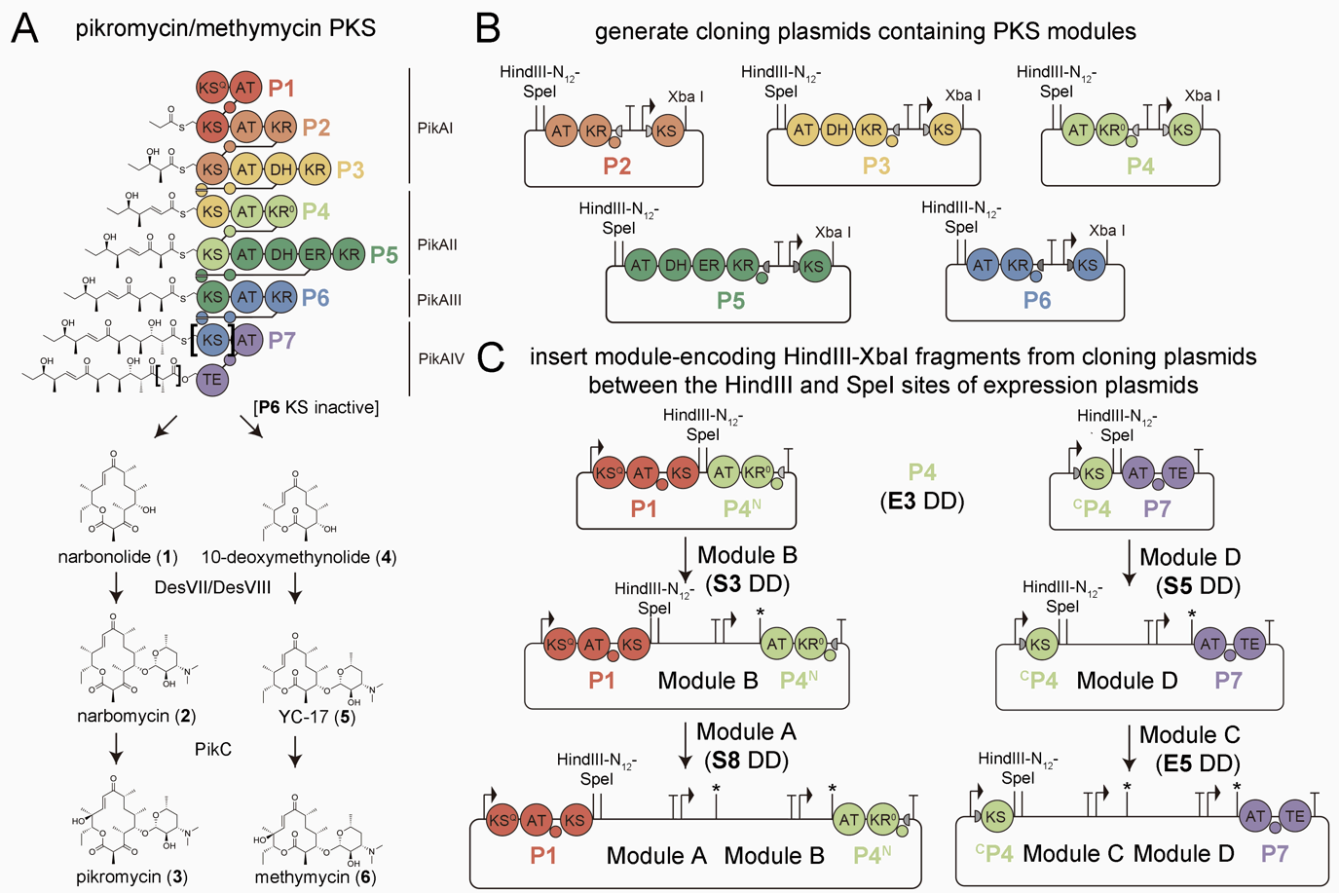
Pikromycin PKS and 2-plasmid platform for constructing engineered PKSs. **a**, Pikromycin/methymycin biosynthetic pathway (PKS colored by updated modules). **b**, Construction of the BioBrick-like units encoding pikromycin modules. T7 promoters and terminators are shown. **c**, The 2-plasmid system enables the BioBrick-like assembly of PKSs such as **P1-A-B-P4-C-D-P7**. Docking domains (DDs) are specific for each position in the constructed synthase. The ligation of HindIII-XbaI fragments into the HindIII-N_12_-SpeI insertion sites yields XbaI/SpeI scars (*) encoding 2 serines at module boundaries.

Here, we describe a 2-plasmid system that enables the expression of a refactored pikromycin synthase in *E. coli* as well as the combinatorial biosynthesis of new macrolactones and macrolide antibiotics (Fig. 1)^17^. One plasmid encodes the 1st pikromycin module (**P1**) and the upstream portion of the 4th module (^**N**^**P4**), while the other plasmid encodes the downstream portion of the 4th module (^**C**^**P4**) and the 7th module (**P7**). Module-encoding inserts can be sequentially ligated into both plasmids. The incorporation of **P2**, **P3**, **P5**, and **P6** into this system yielded a functional, refactored pikromycin synthase in *E. coli* K207–3, comprised of 6 polypeptides rather than the 4 natural ones (PikAI-PikAIV)^18^. Docking domain optimization and expression of the editing thioesterase PikAV improved narbonolide (**1**) production 2.9-fold to 85 mgL^−1^ in shake flasks. Replacing modules with others from the pikromycin and spinosyn synthases yielded new narbonolide derivatives. Deleting, inserting, and mutating modules also resulted in new derivatives, including those of the methymycin precursor 10-deoxymethynolide (10-dml, **4**). The expression of enzymes that biosynthesize desosamine, modify macrolactones with it, hydroxylate the resulting macrolides, and confer macrolide antibiotic resistance to *E. coli* enabled the generation of narbomycin (**2**), pikromycin (**3**), YC-17 (**5**), and methymycin (**6**), as well as 6 derivatives thereof. Knocking out pathways that compete with desosamine biosynthesis with CRISPR/Cas9 increased narbomycin production to 37 mgL^−1^. A new narbomycin derivative (**17**), resulting from the replacement of **P3** by **P5**, was purified, characterized, and observed to possess antibacterial activity.

## RESULTS

### Construction and evaluation of the 2-plasmid system

Plasmids p**P1**-^**N**^**P4** and p^**C**^**P4-P7** were generated through Gibson assembly using pCDF-1b and pET28b, respectively (Fig. 1 and Supplementary Figs. 1–2). Synthetic DNA encoding docking domain motifs (^C^DD and ^N^DD) from the 3rd module of the erythromycin synthase, **E3**, was inserted to help noncovalently connect ^**N**^**P4** and ^**C**^**P4**^19^. A HindIII-N_12_-SpeI insertion site was placed between the regions encoding **P1** and ^**N**^**P4** as well as ^**C**^**P4** and **P7** to enable the sequential insertion of fragments encoding modules **A**–**D** and generate synthases such as **P1-A-B-P4-C-D-P7**. The ^C^DD/^N^DD pairs used for **A**, **B**, **C**, and **D** are from the **S8**, **S3**, **E5**, and **S5** modules of the spinosyn and erythromycin synthases, respectively. The insertion of the DNA encoding a module preserves the HindIII-N_12_-SpeI insertion site and creates a downstream XbaI/SpeI scar that appears on the polypeptide level as 2 serines in a permissive loop at the updated module boundary^20,21^. The cloning plasmids that maintain the updated modules have been previously described^22^. Briefly, they were constructed by inserting synthetic DNA between the HindIII and XbaI sites of pUC19 to introduce a T7 promoter, a *lac* operator, a ribosome binding site, a T7 terminator, restriction sites for importing DNA fragments encoding the upstream and downstream portions of a module (SpeI/BmtI and MfeI/XbaI), and DNA encoding docking domain motifs to aid the noncovalent assembly of the module (Supplementary Fig. 3).

**Figure 2:**
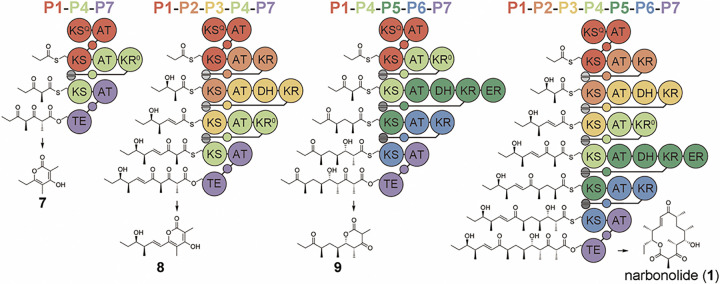
Evaluation of the 2-plasmid system and narbonolide biosynthesis by refactored pikromycin PKS. Expected products are observed from **P1-P4-P7**, **P1-P2-P3-P4-P7**, **P1-P4-P5-P6-P7**, and **P1-P2-P3-P4-P5-P6-P7**.

To test the 2-plasmid system, p**P1**-^**N**^**P4** and p^**C**^**P4**-**P7** were transformed into *E. coli* K207–3^18^. This engineered strain harbors the *Bacillus subtilis* phosphopantetheinyl transferase Sfp and *Streptomyces coelicolor* propionyl-CoA carboxylase to respectively activate ACP domains and help convert propionate supplied to the media into (2*S*)-methylmalonyl-CoA. Transformed cells were cultured in polyketide production media, and the anticipated product of **P1**-**P4**-**P7**, triketide pyrone **7**, was identified by LC/MS (high-resolution used throughout) of the media extract (Fig. 2a and Supplementary Fig. 4). Plasmids p**P1**-**P2**-**P3**-^**N**^**P4** and p^**C**^**P4**-**P5**-**P6**-**P7** were then constructed and respectively co-transformed with p^**C**^**P4**-**P7** and p**P1**-^**N**^**P4**. Pentaketides **8** and **9**, the anticipated products of **P1**-**P2**-**P3**-**P4**-**P7** and **P1**-**P4**-**P5**-**P6**-**P7**, were identified by LC/MS of the media extracts (Figs. 2b-c and Supplementary Figs. 5–6). Plasmids p**P1**-**P2**-**P3**-^**N**^**P4** and p^**C**^**P4**-**P5**-**P6**-**P7** were then co-transformed. The media extract contained a compound with the mass expected for narbonolide (**1**) (Fig. 2d and Supplementary Fig. 7). It was purified by silica gel chromatography and semi-preparative HPLC, and its identity was confirmed by NMR (Supplementary Figs. 8–10)^23^.

### Optimizing narbonolide production from the refactored synthase

Fermentation conditions were identified (10 d at 16°C with 60 mM sodium propionate) that yield a narbonolide titer of 28.9 mgL^−1^ (Supplementary Fig. S11). LC/MS analysis of the media extract revealed that shunt products (the products expected of **P1-P2-P7, P1-P4-P7, P1-P6-P7, P1-P5-P6-P7, P1-P2-P3-P4-P7, P1-P2-P5-P6-P7**, and **P1-P4-P5-P6-P7**) were also generated at lower levels (Supplementary Fig. 12a)^22^. We previously hypothesized such shunt products can be produced due to ACPs not being sufficiently restrained by their downstream docking domain motifs^22^. In the refactored synthase, the ^C^DD/^N^DD pairs of **P3**, **P5**, and **P6** come from **S3**, **E5**, and **S5**, respectively. Thus, replacing these with their natural docking domain motifs could result in more restrained ACPs, fewer shunt products, and an increased narbonolide titer. The DNA fragments encoding the native ^C^DD/^N^DD pairs for **P3**, **P5**, and **P6** were inserted into the **P3**, **P5**, and **P6** cloning plasmids and the natural versions of **P3**, **P5**, and **P6** were independently inserted into the refactored pikromycin synthase. This increased narbonolide production 1.1-, 1.3-, and 1.2-fold, respectively, and the combined replacement of natural **P3**, **P5**, and **P6** increased narbonolide production 1.4-fold to 40.2 mgL^−1^ (Supplementary Fig. 12b). Shunt product titers from synthases harboring the natural docking domains were slightly lower (Supplementary Fig. 12a).

Next, the effect of the editing thioesterase PikAV on the refactored system was investigated. This TEII is thought to hydrolyze acetyl or propionyl groups inappropriately bound to ACPs formed by untimely, KS-mediated decarboxylations of malonyl and methylmalonyl extender units^24^. Within *S. venezuelae*, the overexpression, but not the inactivation, of PikAIV has been observed to decrease pikromycin production^24,25^. However, for other PKS-containing gene clusters the inactivation of TEII has resulted in decreased polyketide production^26–29^. Hypothesizing that some level of PikAV would improve the activity of the refactored pikromycin PKS in *E. coli*^30–32^, PikAV expression plasmids were constructed^33^. A single copy of *pikAV* on a pACYC vector was observed to maximally increase narbonolide production (2.1-fold to 84.6 mgL^−1^)(Supplementary Fig. 13).

### Module-swapping to yield narbonolide derivatives

**Figure 3:**
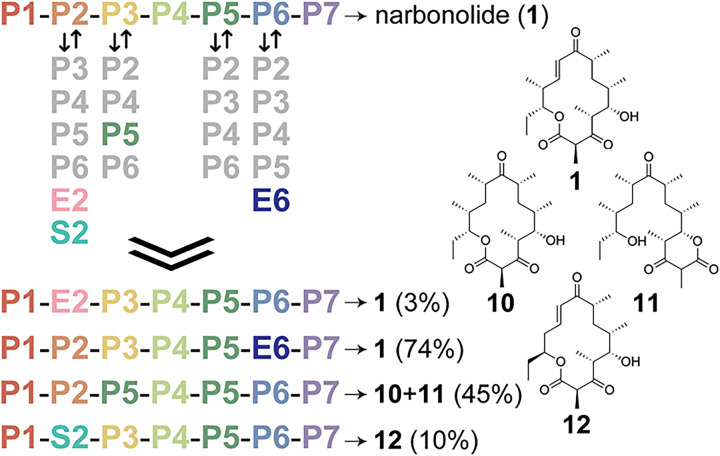
Module swapping of the refactored pikromycin PKS. While 16 swaps with pikromycin modules were attempted, only the replacement of **P3** with **P5**yielded an active PKS. This synthase generates anticipated macrolactone **10**as well as d-lactone **11**. Swapping **P2** and **P6** for **E2**and **E6** (from the erythromycin PKS), respectively, yielded hybrid synthases that generate narbonolide (**1**). Swapping **P2** for **S2**(from the spinosyn PKS) yielded a hybrid synthase that generates macrolactone **12**. Titers are reported in comparison to that of narbonolide by the refactored pikromycin PKS.

With the refactored pikromycin synthase in hand, module-swapping experiments were performed (Fig. 3). Modules **P2**, **P3**, **P5**, and **P6** were replaced with each of the other extension modules of the pikromycin synthase. Of the 16 module-swapped PKSs, only **P1-P2-P5-P4-P5-P6-P7** produced its expected macrolactone (**10**), as confirmed by LC/MS and NMR (Fig. 3 and Supplementary Figs. 14–15). Interestingly, this synthase produced an equivalent quantity of d-lactone **11**, as determined by LC/MS and its absorbance spectrum (Supplementary Fig. 14). The overall heptaketide titer from **P1-P2-P5-P4-P5-P6-P7** (**10 + 11**) is 45% that of the narbonolide titer from **P1-P2-P3-P4-P5-P6-P7**.

To evaluate the compatibility of the refactored pikromycin PKS with modules from other PKSs, **P2** and **P6** were respectively replaced by **E2** and **E6**, erythromycin modules that conduct the same chemistry (Fig. 3). The hybrid synthases **P1-E2-P3-P4-P5-P6-P7** and **P1-P2-P3-P4-P5-E6-P7** respectively produce 3% and 74% the narbonolide titer of **P1-P2-P3-P4-P5-P6-P7** (Supplementary Figs. 16–17). The hybrid synthase **P1-S2-P3-P4-P5-P6-P7** (with **S2** from the spinosyn synthase replacing **P2** of the refactored pikromycin synthase) was also constructed and observed to produce a compound with the mass expected for narbonolide derivative **12** at 10% the narbonolide titer of **P1-P2-P3-P4-P5-P6-P7** and no d-lactone (Fig. 3 and Supplementary Fig. 18).

### Accessing hexaketide and octaketide derivatives

**Figure 4:**
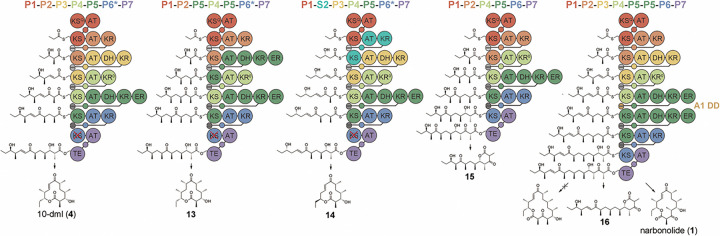
Construction of hexaketide and octaketide synthases. Hexaketide synthases P1-P2-P3-P4-P5-P6*-P7, P1-P2-P5-P4-P5-P6*-P7, P1-S2-P3-P4-P5-P6*-P7, and **P1-P2-P4-P5-P6-P7**, respectively produce **4** (10-dml), **13**, **14**, and **15**. The asterisk (*) next to the **P6** KS indicates the C->A mutation. The octaketide synthase **P1-P2-P3-P4-P5-P5-P6-P7** does not produce the anticipated octaketide macrolactone but does produce octaketide d-lactone **16** and twice as much heptaketide macrolactone **1** (narbonolide). Docking domain (DD) motifs from **A1** of the amphotericin synthase were used for the upstream **P5**.

Within *S. venezuelae*, the heptaketide narbonolide and the hexaketide 10-dml are produced as intermediates in the biosynthesis of pikromycin and methymycin, respectively^17^. Hexaketide production results from an alternate translation start site for PikAIV that inactivates the **P6** KS^34^. To produce 10-dml (**4**) in *E. coli*, a mutagenic approach was employed whereby the **P6** KS reactive cysteine is replaced with an alanine (Figs. 1a and 4a)^35–37^. As confirmed by LC/MS and NMR, the mutated synthase P1-P2-P3-P4-P5-P6*-P7 selectively produces 10-dml with a titer 1.5-fold greater than the narbonolide titer from **P1-P2-P3-P4-P5-P6-P7** (Fig. 4a and Supplementary Figs. 19–22). P1-P2-P5-P4-P5-P6*-P7 and P1-S2-P3-P4-P5-P6*-P7 were constructed, and P1-S2-P3-P4-P5-P6*-P7 was observed to produce low levels of compounds with the masses anticipated for 10-dml derivatives **13** and **14** (Figs. 4b-c and Supplementary Figs. 23–24). Hexaketide synthase **P1-P2-P4-P5-P6-P7** was also constructed and observed to produce d-lactone **15** at a titer 3.2-fold greater than the narbonolide titer from **P1-P2-P3-P4-P5-P6-P7** (Fig. 4a and Supplementary Fig. 25). d-Lactonization of **15** was confirmed by NMR (Supplementary Fig. 26–29). Since octaketide macrolide antibiotics like tylosin and josamycin are naturally-occuring, the octaketide synthase **P1-P2-P3-P4-P5-P5-P6-P7** was also constructed (Fig. 4e). A product of the expected mass was observed; however, its absorbance spectrum revealed it to be d-lactone **16** (Supplementary Fig. 26). Interestingly, narbonolide is the major product of this synthase, at a titer 2.1-fold that of **16**.

### Accessing narbomycin, pikromycin, YC-17, methymycin, and derivatives thereof

**Figure 5:**
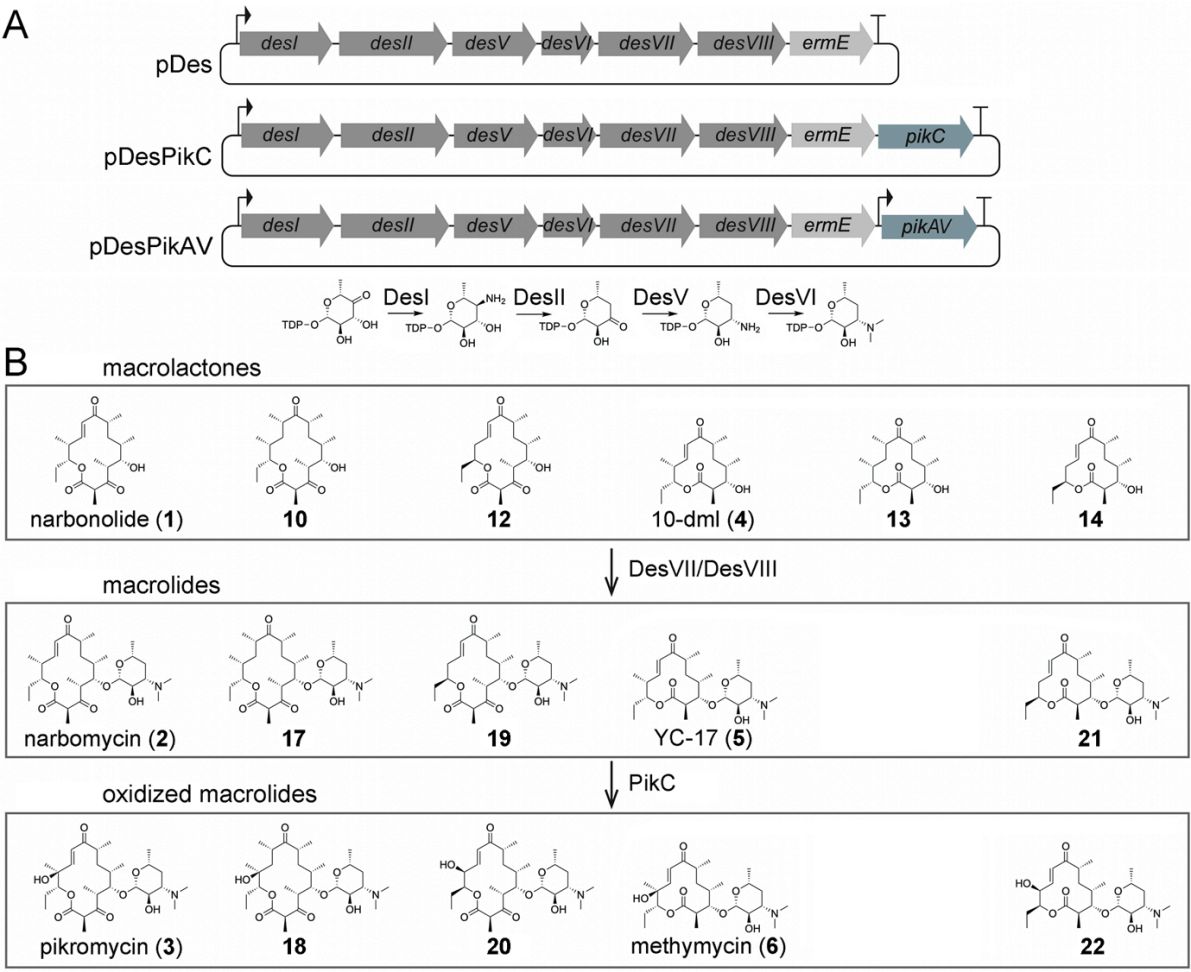
Production of macrolide antibiotics in *E. coli*. **a**, Plasmids pDes, pDesPikC, and pDesPikAV encode desosamine biosynthesis/transfer and macrolide resistance enzymes. Plasmids pDesPikC and pDesPikAV additionally encode the P450 monooxygenase PikC and the editing thioesterase PikAV, respectively. PikAV is controlled by a second T7 promoter in pDesPikAV. **b**, With the exception of **13**, each of the macrolactones in this study is glycosylated by DesVII/DesVIII and hydroxylated by PikC to yield known macrolide antibiotics or derivatives thereof.

The desosamine moiety is critical to the biological activities of macrolide antibiotics^38^. Plasmid pDes was constructed to express the desosamine biosynthetic pathway (DesI, DesII, DesV, and DesVI), the desosamine transferase (DesVII/DesVIII)^39,40^, and the macrolide antibiotic resistance enzyme ErmE (Fig. 5a and Supplementary Fig. 30)^41^. The gene encoding the P450 monooxygenase PikC that converts narbomycin into pikromycin and YC-17 into methymycin, was added to create plasmid pDesPikC^42^. pDes and pDesPikC were transformed into *E. coli* K207–3 harboring PKS expression plasmids. The transformants were cultured in polyketide production media at 16°C for 10 days, and the media extracts were analyzed by LC/MS. *E. coli* K207–3 with pDes completely converted narbonolide to narbomycin (1.3 mgL-1), as confirmed by LC/MS and NMR (Fig. 5a and Supplementary Figs. 31–34)^23^. LC/MS indicates that cells with pDesPikC convert 15% of narbomycin to pikromycin, to provide a pikromycin titer of 0.24 mgL-1 (Fig. 5b and Supplementary Figs. 35–36). Hypothesizing that the low PikC activity is from low protein expression and/or inefficient reduction by *E. coli* proteins, PikC expression was supplemented with plasmid pPikC, which contains another copy of *pikC*^43^. This improved the conversion from narbomycin to 20%, and the pikromycin titer reached 0.31 mgL-1 (Supplementary Fig. 36). P1-P2-P3-P4-P5-P6*-P7 was also expressed with pDes and pDesPikC to respectively yield compounds with mass spectra consistent with **5** (YC-17) and **6** (methymycin) (Fig. 5b and Supplementary Figs. 36–38). NMR confirmed the identity of YC-17 (Supplementary Figs. 39–41).

Next, cells with **P1-P2-P5-P4-P5-P6-P7**, **P1-S2-P3-P4-P5-P6-P7**, P1-P2-P5-P4-P5-P6*-P7, and P1-S2-P3-P4-P5-P6*-P7 were transformed with pDes. While the desosaminylated macrolide anticipated from P1-P2-P5-P4-P5-P6*-P7 was not observed by LC/MS, the desosaminylated macrolides **17**, **19**, and **21** were observed from the other synthases without traces of their respective aglycone precursors **10**, **11**, and **14** (Fig. 5b and Supplementary Figs. 42–44). Narbomycin derivative **17** was isolated and characterized by NMR (Supplementary Figs. 45–47). The addition of PikC through pDesPikC + pPikC yielded small quantities of compounds that yield LC/MS data consistent with oxidized macrolides **18**, **20**, and **22** (Fig. 5b and Supplementary Figs. 48–50).

### Metabolic engineering boosts titers of macrolide antibiotics and their derivatives

**Figure 6:**
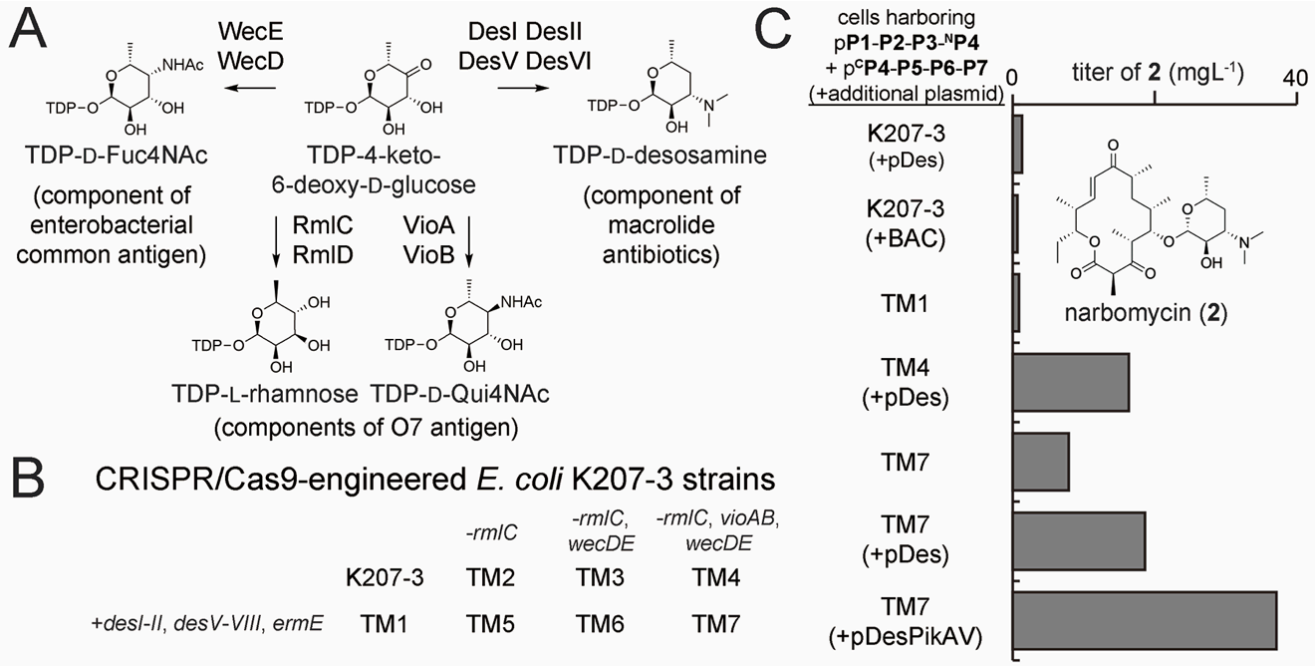
CRISPR/Cas9 engineering helps boost titers of macrolide antibiotics. **a**, Biosynthetic pathways in *E. coli* compete with desosamine biosynthesis for TDP-4-keto-6-deoxy-d-glucose. **b**, The TM1 strain was engineered by inserting the desosamine biosynthesis/transfer genes into the *E. coli* K207–3 genome. The TM2-TM4 and TM5-TM7 strains were engineered through sequentially inactivating *rmlC*, *wecD*/*E*, *vioA*/*B* in *E. coli* K207–3 and TM1, respectively (Supplementary Table 1). **c**, Narbomycin production is reported for engineered *E. coli* strains transformed with p**P1-P2-P3-**^**N**^**P4** & p^**C**^**P4-P5-P6-P7**, encoding the refactored pikromycin synthase, as well as an additional plasmid or BAC that encodes enzymes from the pikromycin biosynthetic pathway (Fig. 5a). TM7 cells with p**P1-P2-P3-**^**N**^**P4**, p^**C**^**P4-P5-P6-P7**, and pDesPikAIV yield a narbomycin (**2**) titer of 37.1 mgL^−1^.

The low titers of narbomycin, pikromycin, YC-17, methymycin, and their derivatives hampers investigations of their structures and antibacterial activities. Since no aglycones were observed from *E. coli* K207–3 generating these macrolides, we hypothesized that the overexpression of the relatively small proteins from pDes, pDesPikC, and pDesPikAV outcompeted PKS expression. Thus, the operon from pDes was transferred to the low copy number (1–2) bacterial artificial chromosome (BAC) pMKBAC02^44^. While narbonolide was completely converted to narbomycin, the narbomycin titer was 69% of that cells transformed with pDes (Fig. 6). The operon was also integrated into the *E. coli* K207–3 genome with CRISPR/Cas9 to yield TM1 cells (Supplementary Fig. 51). Although TM1 cells expressing the refactored pikromycin sythase completely convert narbonolide to narbomycin, the productivity is still only 52% that of *E. coli* K207–3 transformed with pDes.

In a previous study, pathways that utilize TDP-4-keto-6-deoxy-d-glucose were knocked out of *E. coli* K207–3, and the resulting strains, transformed with plasmids encoding desosamine biosynthesis/transfer genes, were observed to more efficiently add desosamine to supplied macrolactones^45^. TDP-4-keto-6-deoxy-d-glucose is not only a precursor in the biosynthesis of the pikromycin monosaccharide, TDP-D-desosamine, but also of the *O*-specific polysaccharide chain of the lipopolysaccharide and the glycolipid on the cell surface of *E. coli* (Fig. 6a)^45–47^. Thus, *rmlC*, *wecD*, *wecE*, *vioA*, and *vioB* were inactivated by CRISPR/Cas9 in both *E. coli* K207–3 and TM1 cells to yield TM4 and TM7 cells, respectively (as well as intermediate strains TM2, TM3, TM5, and TM6) (Fig. 6b and Supplementary Fig. 52). TM4 and TM7 cells, transformed with the plasmids encoding the refactored pikromycin synthase (and pDes for TM4 cells), produce 24- and 12-fold higher narbomycin titers than TM1 cells, respectively (Fig. 6c). TM7 cells transformed with the plasmids encoding the refactored pikromycin synthase and pDesPikAV produce a 55-fold higher narbomycin titer than TM1 cells (37.1 mgL^−1^). Co-transforming TM7 cells with PKS expression plasmids and pDesPikAV or pDesPikC was also observed to boost the titers of other desosaminylated macrolides (Supplementary Table 4).

### Antibacterial activity

Following purification and NMR characterization, narbomycin (**2**), YC-17 (**5**), and **17** were evaluated for antibacterial activity against *Bacillus subtilis 168* (Supplementary Fig. 53). Commercial pikromycin (**3**) was used as a positive control. The minimal inhibitory concentration (MIC) values for **3**, **2**, **5**, and **17** were measured to be 3, 6, 50, and 200 μM, respectively^48,49^.

## DISCUSSION

Since the erythromycin PKS was sequenced in 1990, scientists have been trying to harness the synthetic power of this and other modular PKSs for the production of designer polyketides and new medicines^11,12^. As *E. coli* is more genetically tractable than the natural erythromycin producer *Saccharopolyspora erythraea*, a major goal was realized in 2001 when the erythromycin skeleton, 6-deoxyerythronolide B (6-dEB), was produced by *E. coli*^50^. In 2010, production of the macrolide antibiotic erythromycin A by *E. coli* was reported^51^.

Next to the erythromycin synthase, the pikromycin synthase is the most studied modular PKS^17,52,53^. This is the first report of the reconstitution of the pikromycin synthase in *E. coli* to yield narbonolide as well as the macrolide antibiotics narbomycin and pikromycin. The pikromycin pathway has several advantages over the erythromycin pathway: 1) Fewer tailoring enzymes are needed. The erythromycin pathway requires 6 plasmids, which are not stably maintained by cells containing them. 2) Macrolide antibiotics such as narbomycin and YC-17 can be accessed without P450 enzymes, which are not very active in *E. coli*. The erythromycin pathway contains 2 P450 enzymes, with EryF performing a requisite, first tailoring step. 3) Higher yields of narbomycin (37 mgL^−1^) can be generated compared to erythromycin A (~ 1 mgL^−1^). 4) The methymycin/pikromycin pathway is more versatile, with the PKS having evolved to generate both a hexaketide and a heptaketide and DesVII/DesVIII having evolved to transfer d-desosamine to these 12- and 14-membered macrolactones. 5) Furthermore, the BioBricks-like platform developed here for the refactored pikromycin synthase facilitates the modular synthesis of new macrolide antibacterials through the combinatorial construction of PKSs.

Both the recently reported 1-plasmid system and the 2-plasmid system described here for PKS expression in *E. coli* rely on heterologous docking domain pairs to noncovalently connect the N-terminal and C-terminal portions of modules, which are expressed on separate polypeptides^16,54,55^. Since the current study employs as many as 6 heterologous docking domain pairs per synthase, we compared the performances of heterologous pairs with their natural counterparts. The combined replacement of the **S3**, **E5**, and **S5** docking domain pairs with the docking domains pairs native to **P3**, **P5**, and **P6** improved narbonolide production by 40% (Supplementary Fig. 12). This difference may reflect some degree of co-evolution between docking domains pairs and the modules which they are associated^9,10^. While maximal titers may not be important when first accessing new synthases, native docking domain pairs may be subsequently employed to optimize synthase productivity.

The most significant impediment to combinatorially engineering the refactored pikromycin PKS is KS gatekeeping. While the use of the updated modules ensures compatability of KSs with the a- and b-substituents they are presented, it does not ensure compatability with substituents beyond the b-carbon. Comprehensive module swapping experiments with the pikromycin modules showed that only 1 out of the 16 singly-swapped synthases in which **P2**, **P3**, **P4**, **P5**, or **P6** replaces **P2**, **P3**, **P5**, or **P6** in the refactored pikromycin synthase yields its expected product. That **P1-P2-P5-P4-P5-P6-P7** is functional is consistent with our studies with the 1-plasmid system in which **P5** was observed to function downstream of **P1-P2** in **P1-P2-P5-P7**, **P1-P2-P5-P5-P7**, and **P1-P2-P5-P6-P7**^16^. In these synthases, the **P5** KS accepts a triketide with the same substituents and stereochemistries as in its native substrate at the a-, b-, and g-positions and seems to be tolerant of the differences downstream. However, such differences are not always tolerated by this KS. In the octaketide synthase **P1-P2-P3-P4-P5-P5-P6-P7**, the downstream **P5** KS is presented the same substituents and stereochemistries as in its native substrate at the a-, b-, and g-positions, yet two-thirds of the pentaketide intermediates skip the downstream **P5** module, likely due to low flux through its KS. Thus, even **P5** KS, the most promiscuous of the pikromycin KSs, shows a significant preference for its natural substrate. We are currently experimenting with rapamycin modules, since their KSs have demonstrated greater tolerance to unnatural substrates^16,56,57^.

Our results suggest some level of co-evolution between the upstream modules of synthases. As all of the interactions between acyl-ACPs and their downstream KSs are native in **P1-E2-P3-P4-P5-P6-P7**, KS gatekeeping does not account for its poor activity. This hybrid synthase may be suboptimal due to poor intermodular interactions (*i.e*., upstream KSs with downstream ACPs); however, the robust activity of **P1-P2-P3-P4-P5-E6-P7** suggests this is not a general phenomenon. The specialization of at least the upstream KS domains of PKSs for receiving short substrates indicates that upstream modules may evolutionarily co-migrate^16^. Disruption of these multimodular collaborations could largely explain why **E2** and **S2** exhibit low activity between **P1** and **P3** in **P1-E2-P3-P4-P5-P6-P7** and **P1-S2-P3-P4-P5-P6-P7**.

Some engineered PKSs in this study produce a d-lactone instead of, or in addition to, a macrolactone (**P1-P2-P4-P5-P6-P7** produces **15**, **P1-P2-P5-P4-P5-P6-P7** produces **11** in addition to **10**, **P1-P2-P3-P4-P5-P5-P6-P7** produces **16** in addition to **1**) (Figs. 3 and 4d-e and Supplementary Figs. 14, 25–26). Biochemical and structural studies of the pikromycin TE have revealed that functional groups installed by the first 4 pikromycin modules (**P1**–**P4**) aid in the macrocyclization of the natural hexaketide and heptaketide intermediates^58–60^. A crystal structure of a version of the pikromycin TE in which 2,3-diaminopropionate replaces the catalytic serine and is connected through an amide linkage to the heptaketide intermediate shows complementarity between active site pockets and the C8 and C12 methyl groups as well as a hydrogen bond between the side chain hydroxyl group of Thr1125 and the C9 enone oxygen^60^. These interactions help position the l-oriented C13 hydroxyl group for nucleophilic attack on the C1 carbonyl; however, the relative contributions of these interactions and those at the ACP/TE interface to macrocylization remain unclear. In the context of PikAIV, the pikromycin TE can macrocyclize a heptaketide intermediate in which the ketone is replaced by a hydroxyl group^59^. In this study, the production of macrolactones **10** and **13** by **P1-P2-P5-P4-P5-P6-P7** and P1-P2-P5-P4-P5-P6*-P7, respectively, demonstrates that the double bond is also not required for macrocyclization. Also in this study, the production of macrolactones **12** and **14** by **P1-S2-P3-P4-P5-P6-P7** and P1-S2-P3-P4-P5-P6*-P7, respectively, demonstrates that the nucleophilic hydroxy group does not need to be l-oriented^61^. As combinatorial modular syntheses of macrolide antibiotics are explored, more will be learned about the tolerance of pikromycin TE. If it is a significant bottleneck, promiscuity-enhancing mutations, such as the replacement of the reactive serine by cysteine, will be introduced^62^.

The described platform provides a means to study mysterious assembly line phenomena. For example, it has been debated whether the full pikromycin PKS produces the hexaketide 10-dml or not. In this study, no 10-dml was detected from **P1-P2-P3-P4-P5-P6-P7**. However, the single cysteine-to-alanine point mutation to the **P6** KS renders P1-P2-P3-P4-P5-P6*-P7 an efficient producer of 10-dml. Why **P6** ACP does not transfer the hexaketide intermediate to TE in **P1-P2-P3-P4-P5-P6-P7** is not clear. One hypothesis is that KSs are dimeric when acylated by their polyketide intermediate but monomeric in their absence and that the monomeric state enables the **P6** ACP and TE to approach one another. **P1-P2-P3-P4-P5-P5-P6-P7** may predominantly produce narbonolide due to a similar module-skipping phenomenon that can be investigated through the platform.

Many opportunities exist to boost the titers of polyketides and macrolide antibiotics from *E. coli*. Genomic deletion of a putative propionyl CoA:succinate CoA transferase, *ygfH*, increased the titer of erythromycin A 7-fold^63,64^. The use of bioreactors rather than shake flasks increased the titer of 6-dEB 5-fold^65^. *E. coli* K207–3 has not been optimized for general polyketide production; however, now that this chassis organism can produce macrolide antibiotics, avenues to evolve it for enhanced polyketide production have been opened^18^. For example, the macrolide-sensing transcription factor MphR could be used to help select for *E. coli* K207–3 variants that produce higher titers of narbomycin^66^. To access higher titers of hydroxylated products such as pikromycin and methymycin, PikC activity could be increased through its fusion to a reductase domain^43^.

In summary, we have developed a 2-plasmid BioBrick-like system to assemble PKSs from updated modules and employed it to express a refactored pikromycin PKS in *E. coli*. Through swapping modules and expressing tailoring enzymes from the pikromycin pathway, we modularly synthesized new derivatives of narbonolide, narbomycin, and pikromycin as well as 10-dml, YC-17, and methymycin. Since many strategies beyond those employed here can further boost the yields of macrolide antibiotics, the described platform may be used beyond advancing our understanding of PKS enzymology to access new macrolide antibiotics at preparative levels. Our *E. coli*-based PKS engineering platform is also higher throughput than streptomyces-based platforms^67,68^. We envision it being employed to generate combinatorial libraries of promising polyketide drug leads that will accelerate the medicinal chemistry of these synthetically-challenging compounds.

## Data Availability

All data are available through the Supplementary Information document.
